# Nucleic Acid Preservation Card Surveillance Is Effective for Monitoring Arbovirus Transmission on Crocodile Farms and Provides a One Health Benefit to Northern Australia

**DOI:** 10.3390/v14061342

**Published:** 2022-06-20

**Authors:** Nina Kurucz, Jamie Lee McMahon, Allan Warchot, Glen Hewitson, Jean Barcelon, Frederick Moore, Jasmin Moran, Jessica J. Harrison, Agathe M. G. Colmant, Kyran M. Staunton, Scott A. Ritchie, Michael Townsend, Dagmar Meyer Steiger, Roy A. Hall, Sally R. Isberg, Sonja Hall-Mendelin

**Affiliations:** 1 Medical Entomology, Centre for Disease Control, Public Health Unit, NT Health, Darwin, NT 0811, Australia; medicalentomologyrdh.ths@nt.gov.au (N.K.); allan.warchot@nt.gov.au (A.W.); 2 Public Health Virology, Forensic and Scientific Services, Queensland Health, Coopers Plains, QLD 4108, Australia; jamie.mcmahon@health.qld.gov.au (J.L.M.); glen.hewitson@health.qld.gov.au (G.H.); jean_barcelon05@hotmail.com (J.B.); frederick.moore@health.qld.gov.au (F.M.); 3 Centre for Crocodile Research, Noonamah, NT 0837, Australia; research@crocresearch.com.au; 4 School of Chemistry and Molecular Biosciences, University of Queensland, St. Lucia, QLD 4072, Australia; j.harrison1@uq.edu.au (J.J.H.); agathe.colmant@uq.net.au (A.M.G.C.); roy.hall@uq.edu.au (R.A.H.); 5 Australian Institute of Tropical Health and Medicine, James Cook University, Smithfield, QLD 4878, Australia; kyran.staunton@jcu.edu.au (K.M.S.); scott.ritchie@jcu.edu.au (S.A.R.); michael.townsend@jcu.edu.au (M.T.); dagmar.meyersteiger@my.jcu.edu.au (D.M.S.); 6 Australian Infectious Diseases Centre, University of Queensland, St. Lucia, QLD 4072, Australia

**Keywords:** mosquitoes, Kunjin virus, flaviviruses, surveillance, sentinel chickens, FTA^TM^ cards, virus isolation, saltwater crocodile

## Abstract

The Kunjin strain of West Nile virus (WNV_KUN_) is a mosquito-transmitted flavivirus that can infect farmed saltwater crocodiles in Australia and cause skin lesions that devalue the hides of harvested animals. We implemented a surveillance system using honey-baited nucleic acid preservation cards to monitor WNV_KUN_ and another endemic flavivirus pathogen, Murray Valley encephalitis virus (MVEV), on crocodile farms in northern Australia. The traps were set between February 2018 and July 2020 on three crocodile farms in Darwin (Northern Territory) and one in Cairns (North Queensland) at fortnightly intervals with reduced trapping during the winter months. WNV_KUN_ RNA was detected on all three crocodile farms near Darwin, predominantly between March and May of each year. Two of the NT crocodile farms also yielded the detection of MVE viral RNA sporadically spread between April and November in 2018 and 2020. In contrast, no viral RNA was detected on crocodile farms in Cairns during the entire trapping period. The detection of WNV_KUN_ and MVEV transmission by FTA^TM^ cards on farms in the Northern Territory generally correlated with the detection of their transmission to sentinel chicken flocks in nearby localities around Darwin as part of a separate public health surveillance program. While no isolates of WNV_KUN_ or MVEV were obtained from mosquitoes collected on Darwin crocodile farms immediately following the FTA^TM^ card detections, we did isolate another flavivirus, Kokobera virus (KOKV), from *Culex annulirostris* mosquitoes. Our studies support the use of the FTA^TM^ card system as a sensitive and accurate method to monitor the transmission of WNV_KUN_ and other arboviruses on crocodile farms to enable the timely implementation of mosquito control measures. Our detection of MVEV transmission and isolation of KOKV from mosquitoes also warrants further investigation of their potential role in causing diseases in crocodiles and highlights a “One Health” issue concerning arbovirus transmission to crocodile farm workers. In this context, the introduction of FTA^TM^ cards onto crocodile farms appears to provide an additional surveillance tool to detect arbovirus transmission in the Darwin region, allowing for a more timely intervention of vector control by relevant authorities.

## 1. Introduction

Habitats containing permanent water sources that support mosquito breeding and natural vegetation and that provide sanctuary to wading birds are ideal for the transmission of mosquito-borne viruses. This type of environment is common in parts of tropical northern Australia including the Darwin region of the Northern Territory. Such habitats can be found on saltwater crocodile farms.

In 2016, the Kunjin strain of West Nile virus (WNV_KUN_), belonging to the genus Flavivirus and the family Flaviviridae, was detected in the skin lesions of farmed saltwater crocodiles (*Crocodylus porosus*) in the Northern Territory (NT) of Australia [1]. During the tanning process, these lesions caused by WNV_KUN_ lead to an unevenly structured and dyed skin surface. As a result, crocodile skins found with these lesions are rejected, effecting extensive economic losses for the Australian industry [2].

The mosquito-borne WNV_KUN_ is endemic in northern Australia and is occasionally associated with non-fatal cases of encephalitis in humans. However, the strain of WNV_KUN_ infecting crocodiles is of special interest since it is genetically similar to WNV_KUN_ NSW2011 [2], which was responsible for a large outbreak of fatal equine encephalitis in south-eastern Australia in 2011 [3,4]. The equine virus was shown to be a newly emerged strain that was more neuro-invasive in animal models of the WNV_KUN_ disease, suggesting changes in the epidemiology and ecology of the virus [5].

WNV_KUN_ is predominantly transmitted by the mosquito vector *Culex annulirostris* (Skuse), a ubiquitous species found throughout Australia [6,7,8], while wading birds, especially herons and egrets, are the vertebrate hosts for the virus [9,10]. In the context of human health, the endemic flaviviruses Murray Valley encephalitis virus (MVEV) and Kokobera virus (KOKV) are also transmitted by this vector in Australia [6,11,12,13]. While MVEV causes sporadic cases of severe and fatal encephalitis in humans, infected individuals mostly remain asymptomatic or present with mild symptoms [14]. KOKV infection also occurs occasionally in humans, and may cause acute polyarticular manifestations with febrile illness, taking several months to resolve in some cases [15]. However, these flaviviruses have the potential to emerge as more significant human pathogens, similar to WNV_KUN_, as environmental and societal changes could alter transmission cycles and influence human infections [4,14,15].

While *Cx. annulirostris* is the dominant species found on commercial crocodile farms in the NT, both *Cx. quinquefasciatus* (Theobald) and *Cx. pullus* (Theobald) have been detected breeding on these farms [16,17,18]. *Cx. quinquefasciatus* and *Cx. pullus* have also been implicated in flavivirus transmission. WNV_KUN_ has occasionally been isolated from *Cx. quinquefasciatus*, a species that has been associated with WNV transmission to humans [19,20,21] and in North America with transmission to alligators [22,23]. Johansen et al. (2009) suggested that *Cx. quinquefasciatus* could transmit the virus from birds to humans, acting as a bridge vector [19]. Flaviviruses have also been isolated from field collected *Cx. pullus*, another ornithophilic species possibly maintaining flaviviruses in an enzootic cycle in birds [10,24]. *Cx. gelidus* (Theobald) is of interest as it has shown a high competency for transmitting WNV in laboratory experiments, such as the Australian subtype WNV_KUN_ and to a lesser extent MVEV [25], and an Indian prototype of WNV [26]. However, to date, these flaviviruses have not been isolated from field collected specimens of these species in Australia. 

We established a virus-mosquito surveillance system using passive sentinel mosquito arbovirus capture kits (SMACK traps) with honey-baited nucleic acid preservation cards (FTA^TM^ cards) in order to develop a simple, real-time system to rapidly detect mosquito transmission of WNV_KUN_ on the crocodile farms [27]. In addition, we collected mosquitoes within close proximity using CO_2_-baited encephalitis virus surveillance (EVS) traps to assess the prevalence of vector species and to obtain isolates of WNV_KUN_ for vector incrimination. With a “One Health” approach in mind, we were also interested in the detection of MVEV, which is not known to infect crocodiles, but similar to WNV_KUN_, can cause serious disease in humans [28].

This investigation aimed to establish a logistically convenient system to monitor mosquito-borne flavivirus activity on crocodile farms and develop recommendations for the timely implementation of control strategies. This also allowed us to further define the transmission dynamics of WNV_KUN_ on crocodile farms by comparing data from FTA^TM^ detection with seroconversions to WNV_KUN_ and MVEV in sentinel chicken flocks located in nearby locations around Darwin during the same time period.

## 2. Materials and Methods

### 2.1. Locations of Crocodile Farms

**Figure 1:**
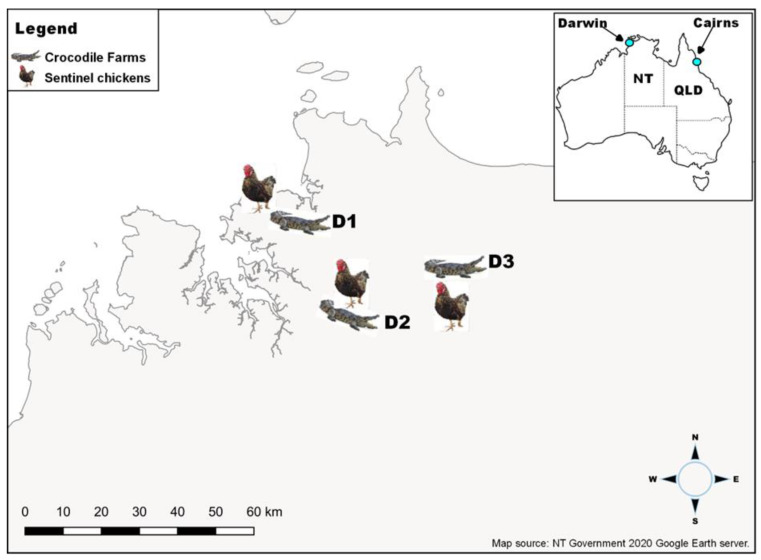
Map of Australia showing the relative locations of Darwin and Cairns in the tropical north of the continent (inset) and crocodile farms in Darwin (D1, D2, and D3) with neighbouring sentinel chicken flocks (main picture).

The investigation was conducted on three commercial crocodile farms, identified as Farms D1, D2, and D3, located in the rural areas of the Darwin region of the NT, Australia (Figure 1), and one farm in the Cairns region (Farm C1) in northern Queensland, Australia (Figure 1). Farms in the Darwin region are in close proximity to freshwater lagoons and wetlands, which are known productive *Cx. annulirostris* breeding areas. The crocodile farm in Cairns (C1) borders on saltwater mangrove environments with mosquitoes around the breeder pens.

### 2.2. Flavivirus Surveillance Using Nucleic Acid Preservation Cards

**Figure 2:**
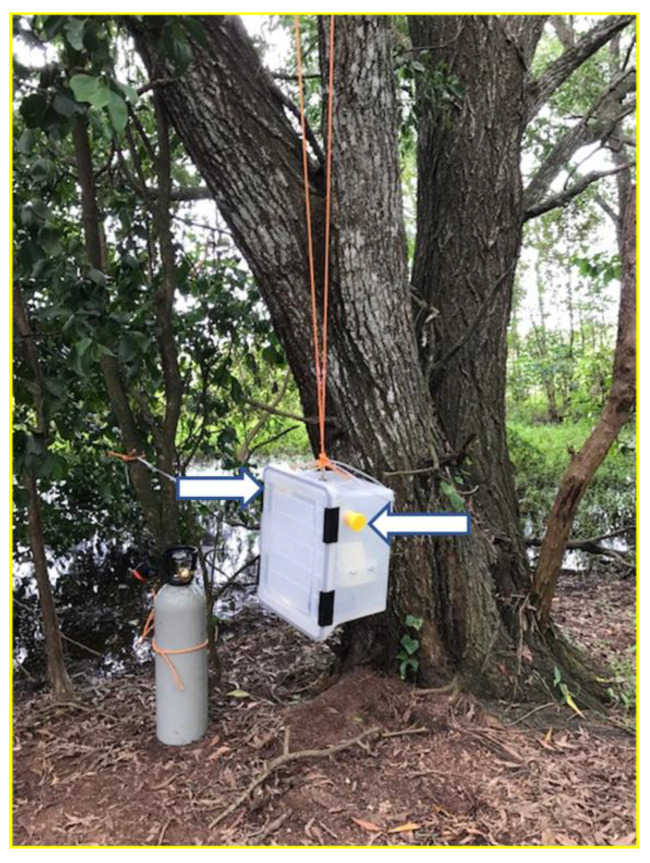
CO_2_-baited SMACK trap housing two honey-baited FTA^TM^ cards (arrows).

To assess the presence of WNV_KUN_ and other flaviviruses at the four crocodile farms, CO_2_-baited SMACK traps were deployed. SMACK has been successfully used in recent years, with honey-soaked nucleic acid preservation cards (FTA^TM^) incorporated into these mosquito traps. The honey attracts trapped mosquitoes, and while feeding they expectorate saliva which also contains any transmissible virus. While the virus is quickly inactivated on the cards, its RNA is preserved. Cards are then sent to the laboratory by normal post and tested for viral RNA by qRT-PCR [27,29,30,31,32] (Figure 2).

In Darwin, two traps were set at each farm in either vegetated areas, assessed to be mosquito harbourage sites, or close to crocodile breeder or grower pens, with the traps serviced and FTA^TM^ cards replaced approximately fortnightly during the high WNV_KUN_ risk period between January and July [33]. Traps were first set on farms D2 and D3 on 21 February 2018 and on farm D1 on 27 February and all were operated until 1 July 2020. During that period, severe cyclone Marcus impacted both D1 traps set between 6 and 22 March 2018. They were replaced with a set running from 7 to 28 March with the following set running just over a week between 28 March and 5 April. During the low WNV_KUN_ risk season, two traps remained operational at D2 and D3 between 2 July and 5 November 2018, before all 6 traps were re-deployed between 5 November 2018 and 1 July 2019. During the second off-season, only 1 trap at D2 was operated between 1 July to 5 November 2019, before starting at D1 on 6 January and at D3 on 7 January 2020 with all 6 traps operational until 1 July 2020. In total, 256 traps were set with 93 in 2018, 87 in 2019, and 76 in 2020, fitted with 2 FTA^TM^ cards per trap (Table S1).

In Cairns, two traps were run from 6 March to 26 June 2018, 13 February to 5 June 2019, and from 7 January to 26 March 2020, totalling 44 traps (16 traps each in 2018 and 2019 and 12 traps in 2020) set at C1 with 2 FTA^TM^ cards per trap (Table S1).

During the fortnightly trap service, mosquito numbers were visually estimated for each trap, then discarded. However, these estimates are not necessarily representative since mosquitoes could escape, or as on a few occasions, ants could find access into traps and eat the mosquitoes. Farms were also routinely sprayed to reduce mosquito numbers. The FTA^TM^ cards from each trap were placed in separate sealable plastic bags and stored at room temperature before being sent to the laboratory at Public Health Virology, Forensic and Scientific Services, Queensland Health, for testing. Surgical gloves were used during handling of the cards to avoid sample contamination.

### 2.3. Processing of FTA^TM^ Cards and Detection of Viral RNA

In the laboratory, cards were kept at ambient temperature but placed on ice as soon as processing began. Methods of elution as described in Hall-Mendelin et al. (2010) [27] were followed with two modifications: the cards were vortexed in molecular grade water to elute nucleic acids prior to extraction on a Qiagen BioRobot Universal System, and QIAamp One-For-All Nucleic Acid Kit (Qiagen, Clifton Hill, VIC, Australia) was used according to instructions. Viral RNA was detected by TaqMan RT-PCR using specific assays for WNV_KUN_ and MVEV. WNV_KUN_ RNA was amplified with the following primers and probe: Primer Kunjin-F AACCCCAGTGGAGAAGTGGA at 900 nM/µL, Primer Kunjin-R TCAGGCTGCCACACCAAA at 900 nM/µL, and Probe Kunjin MGB 6FAM-CGATGTTCCATACTCTGG-MGB at 150 nM/µL [34] (Finger, unpublished). MVEV RNA amplification was performed using MVE-FOR ATCTGGTGYGGAAGYCTCA at 900 nM/µL, MVE-REV CGCGTAGATGTTCTCAGCCC at 900 nM/µL, and MVEV-Probe 6FAM-ATGTTGCCCTGGTCCTGGTCCCT-TAMRA at 200 nM/µL [34]. Detection of both templates was performed in a 20 µL reaction volume in a Rotor-Gene Q real-time PCR cycler (Qiagen, Chadstone, VIC, Australia). Cycling conditions were set as follows: one cycle at 50 °C for 5 min, one cycle at 95 °C for 2 min, and 50 cycles at 95 °C for 3 s and 60 °C for 30 s using the Superscript III Platinum one-step quantitative qRT-PCR system (Invitrogen, Carlsbad, CA, USA) which was used as per the manufacturer’s instructions. Separate synthetic controls for primers and probe of both viruses, and no template controls were included in each Rotor-Gene run [35]. A sample was generally deemed positive with Ct values <40 and negative when Ct values were >40. These assays are NATA accredited.

### 2.4. Sentinel Chickens in Darwin

In order to issue timely public health warnings, sentinel chickens have been the primary method for flavivirus surveillance in the NT since 1992, with seroconversions to WNV_KUN_ and/or MVEV recorded in most years [36], whereas Cairns (QLD) does not operate a sentinel animal program for arbovirus detection.

Four sentinel chicken flocks consisting of 8 to 12 chickens were used, with the Leanyer flock located 7 km from crocodile farm D1, the Beatrice Hill Farm (BHF) flock 10 km from crocodile farm D3, and the Howard Springs and Bees Creek flocks situated 12 km and 0.05 km, respectively, from crocodile farm D2. The Leanyer, Howard Springs, and BHF flocks are in close proximity to extensive wetlands and have been part of the NT flavivirus surveillance program since 1992–1993, while the Bees Creek flock was only established in early March 2020. Chickens were bled monthly during the high risk WNV_KUN_ period (January to June), with an initial baseline bleed in December 2018 and 2019. Samples were tested for antibodies to viruses using standard flavivirus neutralisation tests as described [37]. Bleeding of sentinel chickens was carried out under ethics approval (monitoring sentinel animals for viruses of animal and human health significance number A11033. 100/annum) granted by the Charles Darwin University Research Ethics Committee.

### 2.5. Mosquito Collections

Following detection of WNV_KUN_ on FTA^TM^ cards from SMACK traps, encephalitis virus surveillance (EVS) traps were then set at the same locations on the farms to maximise the chance of collecting mosquitoes carrying WNV_KUN_ for virus isolation, with a total of 22 traps set in 2018 and 17 traps in 2019. 

To maintain the required cold chain for virus isolation work, mosquito traps were transported from site in an insulated container with dry ice before mosquitoes were stored at −80 °C. Mosquito identification to species level was performed on cold tables at the Medical Entomology laboratory in Darwin using taxonomic keys [38,39,40,41,42,43]. Identified mosquito species were stored in vials up to 50 specimens, and blood fed specimens or specimens with mites attached were not processed.

### 2.6. Virus Isolation

Mosquito pools containing up to 50 individuals were shipped to the laboratory on dry ice and then stored at −80 °C until processing for flavivirus isolation. Pools of mosquitoes were then homogenised in 2 mL of medium (Opti-MEM, GIBCO, Life Technologies, Grand Island, NY, USA), supplemented with 3% fetal bovine serum (In Vitro Technologies, Australian origin), antibiotics, and antimycotics (GIBCO, Life Technologies, Grand Island, NY, USA), using one metal bead in a Tissue Lyser II (Qiagen, Hilden, Germany). After centrifugation, supernatants were filtered. We used 0.2 µm size syringe filters for pools containing < 5 mosquitoes and the 0.8/0.2 µm double filter units for larger pools (PALL Corporation, Ann Arbor, MI, USA). Sterile homogenates were inoculated in quadruplet, 50 µL per well, on duplicate 96-well plates coated with a monolayer of C6/36 cells. The cultures were incubated for 7 days at 28 °C, then fixed in cold 20% acetone after removal of supernatants which were stored at −80 °C. Fixed, dried plates were stored at −20 °C. Presence of flaviviruses was detected on the plates with an ELISA using monoclonal antibodies 4G2 (pan flavivirus anti-E) and 4G4 (pan flavivirus anti-NS1) [44]. Briefly, plates were blocked for 1 h with 100 µL per well of blocking buffer, prior to the addition of a cocktail of 4G2 and 4G4, each diluted 1:100 in blocking buffer, at 50 µL per well. After 1h incubation at room temperature, the plates were washed 6 times then anti-mouse IgG HPR conjugate (diluted 1/2000 in blocking buffer) was added at 50 µL per well. After further incubation and washing of plates, TMB substrate (50 µL/well) was added to visualise the reaction, and the reaction stopped with H_2_O_2_ buffer. Optical densities (OD) were measured with a plate reader (TECAN Minilyser Spectra II, Tecan Group Ltd., Maennedorf, Switzerland) at a wavelength of 450 nm and a reference wavelength of 620 nm. OD readings were called positive when they were three × standard deviation higher than the negative samples.

Supernatant from samples reacting in this ELISA were re-inoculated onto fresh C6/36 cultures and incubated for 4 days at 28 °C. Supernatants were collected and plates fixed as described above. A panel of mAbs specific for a range of medically significant flaviviruses previously detected in Northern Australia (WNV_KUN_-specific—3.1112G, 10A1, 3.101C, 3.91D; MVEV-specific—10C6; JEV-specific 989; and KOKV-specific—1C1) were used to identify virus in an ELISA system as described above. The identity of detected virus was confirmed by a KOKV-specific RT-PCR and Sanger sequencing of the amplicon. The reactive sample was also deep sequenced (HiSeq, Illumina, San Diego, CA, USA), using standard methods that we have previously used for sequencing viruses from mosquitoes captured in crocodile farms, published in [2].

### 2.7. Phylogeny

Multiple amino acid sequence alignments of the new KOKV isolate (KOKV_A2019-0110,_ accession number OL347997) and selected flaviviruses were performed with MAFFT v7.388 algorithm, using a scoring matrix of BLOSUM62, a gap open penalty of 1.53, and an offset value of 0.123 [45,46]. FastTree 2.1.5 was used to construct a phylogenetic tree that uses the maximum likelihood approximation method, with optimisation for Gamma20 likelihood selected, while the branch support values were calculated using a Shimodaira-Hasegawa test. Analyses were undertaken within the Geneious 11.1.5 package.

## 3. Results

### 3.1. WNV_KUN_ and MVEV Surveillance on Crocodile Farms

Each trap contained two FTA^TM^ cards; a trap was classified as virus-positive when viral RNA was detected on at least one of the cards. Between Feb 2018 and July 2020, WNV_KUN_ RNA was detected on FTA^TM^ cards in each year with the most detections occurring in the months of January to May (Table 1, Table S1), consistent with the increased mosquito activity during the monsoon season, late wet season, and the start of the dry season [47]. The most frequent WNV_KUN_ activity was detected in March 2019 and April 2020, where traps on all three farms yielded positive cards, often with both cards in the trap positive for the virus, totaling 24 WNV_KUN_-positive and 8 MVEV-positive traps (Table S1).

In 2018, no virus was detected at crocodile farm D1 while WNV_KUN_ RNA was detected at D2 in March and April and at D3 in March, with a total of three traps positive for WNV_KUN_ RNA in the first year (Table 2). In 2019, WNV_KUN_ RNA was detected in February, March, and May at D1; in March, July, and October at D2; and in March at D3, with a total of 11 traps positive for the virus (Table 2). In 2020, WNV_KUN_ RNA was detected in January and again in July at D3, in April at all three crocodile farms, and in May at D2, with a total of 10 positive traps for this year (Table 2).

Detections of MVEV RNA were sporadically spread between April and November over the two and a half years of trapping. Detection was most frequent in 2018, first appearing at D3 in April and in May and then again in November. It was detected once at D2 in July, with a total of four positive traps for MVEV RNA in this year (Table 2). In 2019, MVEV RNA was not detected until July and again in October, both times at D2. This correlated with the sporadic appearance of WNV_KUN_ at D2, with both viruses detected in the same traps (Figure S1 and Table 2). In 2020, MVEV RNA was detected in April on the same cards as WNV_KUN_ at D2 and was found in May on a single card at D3 (Table 2).

WNV_KUN_ RNA was not detected on the Cairns crocodile farm (C1), where it was the only targeted virus.

In Darwin, detections of viral RNA on FTA^TM^ cards on the crocodile farms were compared with sentinel chicken seroconversion to flaviviruses at the Beatrice Hill Farm, the Leanyer, Howard Springs, and Bees Creek flocks (Figure 1). Between February 2018 and June 2020, five seroconversions to WNV_KUN_ were detected: at Beatrice Hill Farm (near D3), a seroconversion was detected on 1 March 2018 and 7 March 2019; two at Howard Springs (near D2) on 13 March 2019; and one at Leanyer (near D1) on 8 January 2020 (Figure 1). Each of the seroconversions in 2018 and 2019 correlated with the detection of WNV_KUN_ RNA on FTA^TM^ cards at nearby farms in the same time period. While there were no positive FTA^TM^ cards at D1 in January 2020 when seroconversion occurred in the nearby sentinel chicken flock at Leanyer, the FTA^TM^ cards were positive at D3 indicating WNV_KUN_ activity in the general Darwin region.

Seroconversions to MVEV were only detected in 2018 (Table 1 and Table S1). At the Beatrice Hill Farm, sentinel chickens seroconverted to MVE on 29 March, and again on 3 May 2018. At Leanyer, one chicken seroconverted on 10 April 2018 and one at Howard Spring on 2 May 2018. The seroconversions at the Beatrice Hill Farm (29 March) correlated with MVEV being detected on cards in traps set on D3 (April-May). Sentinel chicken seroconversion to MVEV at the Beatrice Hill Farm on 29 March was also coincident with MVEV-positive FTA^TM^ cards detected in traps set at the nearby D3 farm (March–April). In the same locations, chicken seroconversion and positive FTA^TM^ cards correlated in April–May.

### 3.2. Virus Isolation

In 2018, 4380 mosquitoes were collected with the catches consisting mainly of *Cx. annulirostris* (62%) but also several other species, such as *Mansonia uniformis*, *Anopheles bancroftii*, *Cx. palpalis*, *Cx. pullus*, *Cq. xanthogaster*, and *Cx. quinquefasciatus*. When these mosquitoes were pooled and processed for virus isolation, no vertebrate-infecting flaviviruses such as MVE or WNV_KUN_ were detected. However, several insect-specific viruses were isolated and will be reported elsewhere (Colmant et al. unpublished data).

**Figure 3:**
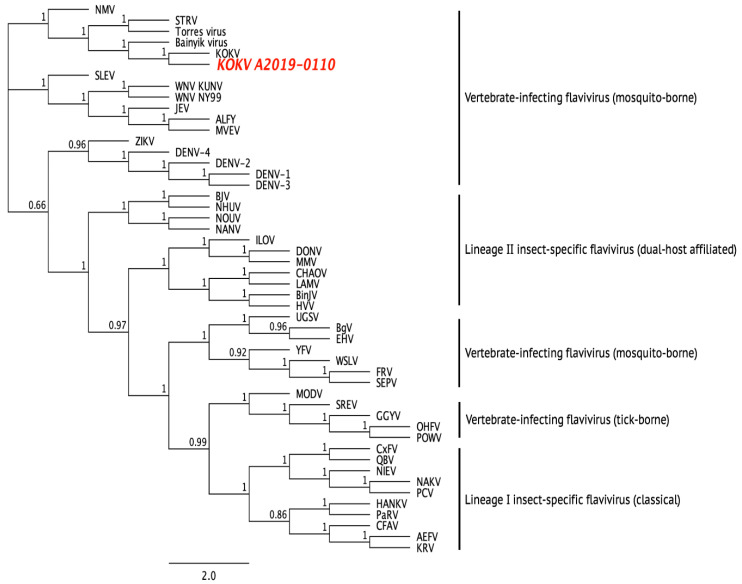
Dendrogram showing phylogenetic relationship between the prototype KOKV, KOKV A2019-0110, and other flaviviruses using a maximum-likelihood model and complete amino acid sequences. Sequences were derived using the following GenBank accession numbers: AEFV AB488408, ALFV AY898809, Bainyik virus KM225264, BgV KU308380, BinJV MG587038, BJV KC496020, CFAV KJ741267, CHAOV JQ308185, CxFV AB262759, DENV-1 U88536, DENV-2 U87411, DENV-3 AY099336, DENV-4 AF326825, DONV NC_016997, EHV DQ859060, FRV KM361634, GGYV DQ235145, HANKV NC_030401, HVV MN954647, ILOV KC734549, JEV NC_001437, KOKV AY632541, KOUV MN057643, KRV AY149905, LAMV KC692068, MMV MF139576, MODV AJ242984, MVEV AF161266, NAKV NC_030400, NANV MF139575, NHUV KJ210048, NIEV JQ957875, NMV KC788512, NOUV EU159426, OHFV AY193805, PaRV KT192549, PCV KC505248, POWV L06436, QBV FJ644291, SEPV DQ837642, SREV DQ235150, STRV KM225263, Torres virus KM225265, UGSV DQ859065, WNV KY229074, WSLV JN226796, YFV X03700, and ZIKV AY632535.

In 2019, 6206 non-blood fed and identifiable mosquitoes from 17 EVS traps were tested for the presence of viruses, including 2620 *Cx. annulirostris*, 79 *Cx. pullus*, 42 *Cx. quinquefasciatus*, and 35 *Cx. gelidus* (Table 3). Other mosquito species tested, totalling 3430, are listed in Table 3. While no WNV_KUN_ or MVEV was isolated, a flavivirus-like isolate was detected in one pool of *Cx.* *annulirostris* mosquitoes (A2019-0110) trapped at D3 on 28 March 2019 (Table 4). When culture supernatants from the cells inoculated with this sample were further passaged onto C6/36 cells and tested with a panel of mAbs specific for medically important flaviviruses, only the KOKV-specific mAb 1C1 was reactive to the fixed inoculated cells. The identity of this virus was confirmed as Kokobera by a KOKV-specific RT-PCR and whole genome sequencing. BLAST analysis of the genome contig derived from sequencing data showed a high degree of similarity with the prototype KOKV virus (accession number NC_009029 [48]). Sequence alignment of the complete ORF of the new isolate further confirmed its identity as a new strain of KOKV (Figure 3).

## 4. Discussion

In this study, we successfully established the FTA^TM^ card surveillance system on crocodile farms in the Northern Territory and north Queensland and detected WNV_KUN_ and/or MVEV transmission on each farm except for north Queensland. As expected, transmission was most prevalent between January to May which is generally considered the period of peak arbovirus activity. We and others have previously validated FTA^TM^ cards as an effective arbovirus surveillance system for several mosquito-borne viruses in a range of environments and locations [27,30,32,49,50,51,52]. However, this was the first report of its use to monitor virus transmission in the context of farmed crocodilians and to guide the frequency and timing of mosquito control strategies (e.g., spraying). Indeed, when compared to a nearby concurrently run program based on the seroconversion of sentinel chickens, transmission was detected more frequently by FTA^TM^ cards on crocodile farms than by sentinel chicken surveillance conducted in the same timeframe. Discrepancies between the two surveillance systems may be attributed to random differences in the transmission frequency between the different locations of the sentinel chicken flocks relative to the crocodile farms within the Darwin region, or to a difference in sensitivity between the two systems. The increased detection of viral transmission on crocodile farms may also reflect the unique transmission dynamic of WNV_KUN_ that can occur directly between farmed crocodilian species, thus providing additional sources of mosquito infection and transmission [2]. However, to date, this has only been demonstrated for alligators. Thus, regular testing of crocodile pen water for viral RNA should also be considered to monitor other avenues of WNV_KUN_ transmission on farms.

Despite evidence of their presence on FTA^TM^ cards on the farms, we failed to isolate WNV_KUN_ or MVEV from mosquitoes trapped in close proximity and within the same time frame of positive FTA^TM^ card detections. This suggested that there was a low prevalence of virus in the mosquito population, consistent with only one of four FTA^TM^ cards usually yielding a positive result on each farm at each transmission event and is further supported by the relatively high Ct scores (>35) that were generally observed. However, while EVS traps were set immediately after the FTA^TM^ cards were reported to be positive for WNV_KUN_ or MVEV RNA, we cannot rule out the possibility that we had already missed a narrow window of transmission by the time the mosquitoes were collected. Reduced overall mosquito numbers from routine mosquito control measures (spraying) may also have reduced our ability to isolate these viruses.

Interestingly, no human cases due to WNV_KUN_ infection were reported during our trapping period and only one case of MVEV infection occurred in early May 2018 (a resident of a remote area of Arnamland in NT, approximately >200 kms East of the Darwin region who was not associated with crocodile farms). 

The isolation of Kokobera virus from a trap set at D3 in 2019 is worthy of further investigation. It is feasible that this flavivirus could infect and cause disease in crocodiles, even though it is considered a relatively benign virus and has only ever been associated with rare cases of a mild febrile illness in humans manifesting as polyarthralgia, headache, and skin lesions [15]. Furthermore, the detection of MVEV RNA on FTA^TM^ cards collected on the farms indicates that, in addition to WNV_KUN,_ this virus could also be transmitted to crocodiles and cause disease. Future studies should be directed at sampling farmed crocodiles and testing for the presence of MVEV- and KOKV- specific antibodies in serum as evidence that these viruses can productively infect these animals.

While WNV_KUN_ and/or MVEV activity was detected in all the investigated years by FTA^TM^ card surveillance on the crocodile farms in the Darwin region, the same surveillance program on farms in Cairns did not detect RNA from WNV_KUN_ on any of the cards collected over the entire study period (cards were not tested for MVEV RNA). This is consistent with our understanding of the epidemiology of WNV_KUN_ and MVEV, which are regularly detected in the region around Darwin in the Northern Territory and the northern and western regions of Cape York Peninsula in Queensland, but rarely detected on the eastern side of Cape York where Cairns is situated [25,29,30].

## 5. Conclusions

In conclusion, we have successfully established the FTA^TM^ card surveillance system on crocodile farms and have shown that it is a reliable indicator of the transmission of WNV_KUN_ and MVEV on farms during times of peak activity around Darwin. This can provide a timely warning to implement control measures to reduce WNV_KUN_ disease in crocodiles by vector control. The early detection of the transmission of these viruses on crocodile farms near Darwin also has important “One Health” implications, allowing more timely intervention of vector control measures to protect residents of the Darwin region from arboviral infections.

## Figures and Tables

**Table 1:** WNV_KUN_- and MVE-positive FTA^TM^ cards from SMACK traps set between February 2018 and July 2020 compared to sentinel chicken seroconversions over the same period. Sentinel chicken program was run January to June.

Croc Farm	Collection Period	Trap ID	Virus Detected (Positive Cards/Cards Set)	Chicken Seroconversions Date; Chicken Farm; Virus
D1	March–December 2018		All Negative	April 2018; LF; MVEV
	January–February 2019	1	WNV_KUN_ (1/2)	
	February 2019	1	WNV_KUN_ (1/2)	
	February–March 2019	1	WNV_KUN_ (2/2)	
		2	WNV_KUN_ (2/2)	
	May 2019	1	WNV_KUN_ (1/2)	
	April 2020	1	WNV_KUN_ (1/2)	January 2020; LF; WNV_KUN_
		2	WNV_KUN_ (2/2)	
D2	March 2018	2	WNV_KUN_ (1/2)	
	March–April 2018	1	WNV_KUN_ (1/2)	May 2018; HSF; MVEV
	June–July 2018	2	MVEV (1/2)	
	February–March 2019	1	WNV_KUN_ (2/2)	
		2	WNV_KUN_ (2/2)	March 2019; HSF; WNV_KUN_
	July 2019	1	WNV_KUN_ (1/2), MVEV (1/2)	
	September–October 2019	1	WNV_KUN_ (2/2), MVEV (1/2)	
	April 2020	1	WNV_KUN_ (2/2), MVEV (1/2)	
		2	WNV_KUN_ (1/2)	
	April–May 2020	1	WNV_KUN_ (1/2)	
		2	WNV_KUN_ (1/2)	
D3	February –March 2018	1	WNV_KUN_ (1/2)	March 2018; BHF; WNV_KUN_
	March–April 2018	2	MVEV (2/2)	March 2018; BHF; MVEV
	April–May 2018	2	MVEV (2/2)	May 2018; BHF; MVEV
	October–November 2018	1	MVEV (1/2)	
	February–March 2019	1	WNV_KUN_ (2/2)	March 2019; BHF; WNV_KUN_
		2	WNV_KUN_ (2/2)	
	January 2020 A	2	WNV_KUN_ (1/2)	
	January 2020 B	2	WNV_KUN_ (2/2)	
	April 2020	1	WNV_KUN_ (1/2)	
	May 2020	2	MVEV (1/2)	
	June–July 2020	2	WNV_KUN_ (1/2)	

D1, D2, and D3 are crocodile farms in rural Darwin, NT; LF Leanyer Farm sentinel chicken flocks near D1; HSF Howard Springs Farm sentinel chicken flocks near D2; BHF Beatrice Hill Farm sentinel chicken flocks near D3.

**Table 2:** Number of SMACK traps set and numbers of virus-positive traps.

	2018			2019			2020		
Farm Location	# of Traps	WNV_KUN_ Pos	MVEV Pos	# of Traps	WNV_KUN_ Pos	MVEV Pos	# of Traps	WNV_KUN_ Pos	MVEV Pos
D1	26	0	0	26	5	0	26	2	0
D2	33	2	1	35	4	2	24	4	1
D3	34	1	3	26	2	0	26	4	1
	93	3	4	87	11	2	76	10	2
C1	16	0	0	16	0	0	12	0	0

D1, D2 and D3 are crocodile farms in rural Darwin, NT. C1 is a crocodile farm in rural Cairns, Qld.

**Table 3:** Mosquito species collected at Darwin crocodile farms in 2019.

Mosquito Species		Number	Sub-Totals
Known/potential WNV_KUN_ vectors	*Culex annulirostris*	2620	
	*Cx. pullus*	79	
	*Cx. quinquefasciatus*	42	
	*Cx. gelidus*	35	2776
Other species	*Mansonia uniformis*	1707	
	*Cx. species*	620	
	*Anopheles bancroftii*	597	
	*Coquillettidia xanthogaster*	413	
	*Cx. squamosus*	67	
	*Cx. bitaeniorhynchus*	9	
	*Cx. hilli*	6	
	*Cx. vishnui group*	3	
	*Aedes kochi*	2	
	*Ae. vigilax*	1	
	*An. powelli*	1	
	*Cx. vishnui*	1	
	*Uranotaenia albescens*	1	
	*Ur. lateralis*	1	
	*Ur. nivipes*	1	3430
	Total		6206

**Table 4:** Virus isolation attempts from *Cx. annulirostris* mosquitoes collected in EVS traps set on Darwin crocodile farms in 2019.

Location	Date Traps Set	No. *Cx. annulirostris* Processed Mosquitoes/Pools	Virus Isolation Positive Pool ID; Virus
D1	5 March 2019	698/16	None
	20 March 2019	93/5	None
D3	28 March 2019	951/21	A2019-0110; KOKV
	12 April 2019	878/19	None
Total		2620/61	1

D1 and D3 are crocodile farms in rural Darwin, NT; KOKV Kokobera virus.

## Data Availability

Data supporting the reported sentinel results can be found at: https://digitallibrary.health.nt.gov.au/prodjspui/bitstream/10137/11788/1/Medical%20Entomology%20Annual%20Report%202019_2020.pdf. Date Accessed: 1 June 2022.

## Supplementary Materials

The following are available online at https://www.mdpi.com/article/10.3390/v14061342/s1, Table S1: Summary of FTA results and sentinel chicken data collected from Darwin region over the course of the study (2018–2019, sentinel chicken program run January–June).
