# Supplementary material for: Nucleic Acid Preservation Card Surveillance Is Effective for Monitoring Arbovirus Transmission on Crocodile Farms and Provides a One Health Benefit to Northern Australia
Source: Viruses. 2022 Jun 20;14(6):1342. doi: 10.3390/v14061342 (PMC9227548; doi:10.3390/v14061342)
Supplement: Supplementary file 1 [file viruses-14-01342-s001.zip › viruses-1733791-supplementary.pdf]

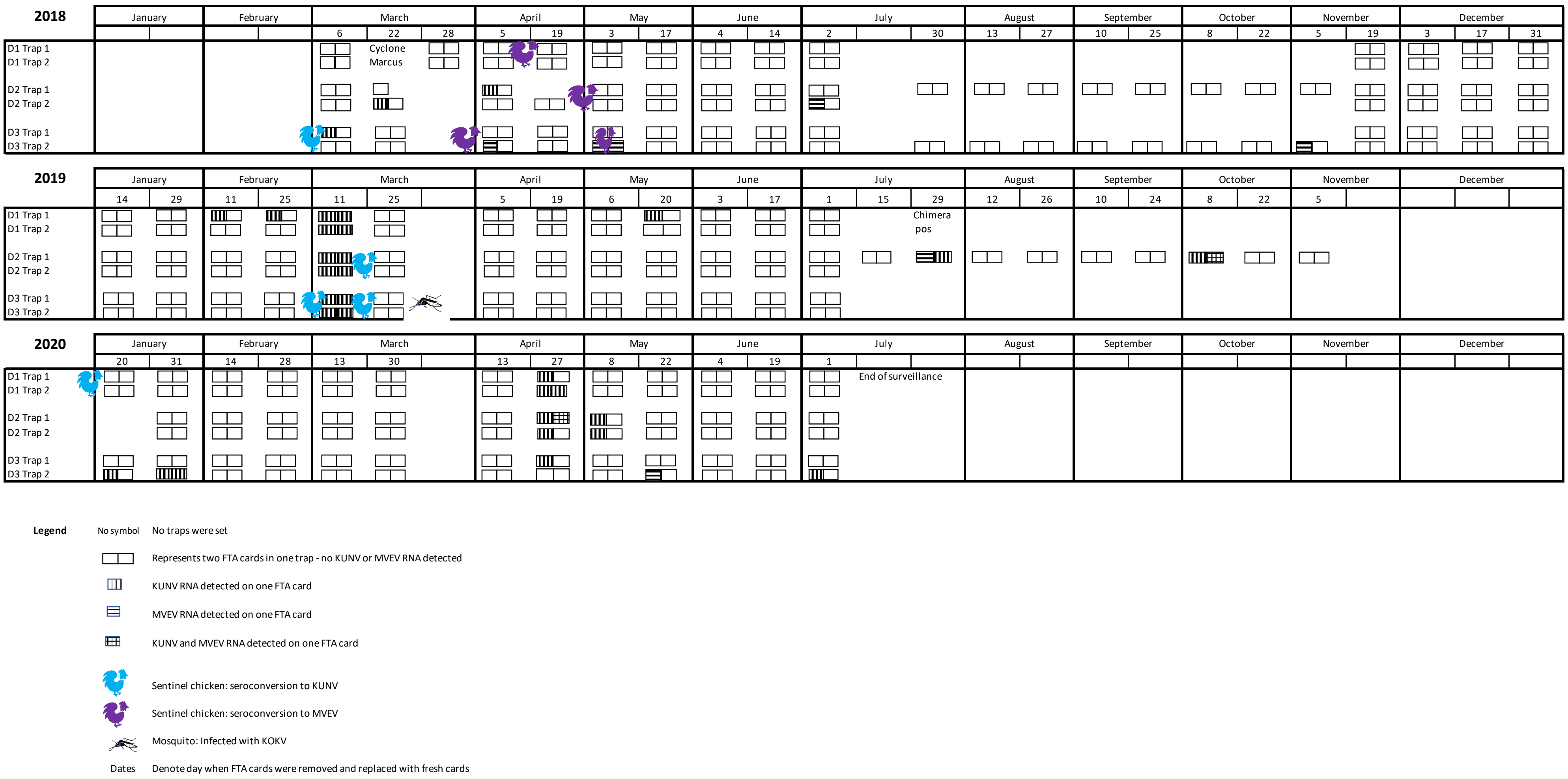
2018
January
February
March
April
May
June
July
August
September
October
November
December
6 22 28 5 19 3 17 4 14 2 30 13 27 10 25 8 22 5 19 3 17 31
D1 Trap 1
D1 Trap 2
D2 Trap 1
D2 Trap 2
D3 Trap 1
D3 Trap 2
Cyclone
Marcus
2019
14 29 11 25 11 25 5 19 6 20 3 17 1 15 29 12 26 10 24 8 22 5
Chimera
pos
2020
20 31 14 28 13 30 13 27 8 22 4 19 1
End of surveillance
Legend
No symbol
No traps were set
Represents two FTA cards in one trap - no KUNV or MVEV RNA detected
KUNV RNA detected on one FTA card
MVEV RNA detected on one FTA card
KUNV and MVEV RNA detected on one FTA card
Sentinel chicken: seroconversion to KUNV
Sentinel chicken: seroconversion to MVEV
Mosquito: Infected with KOKV
Dates
Denote day when FTA cards were removed and replaced with fresh cards
